# The Ability of PVX p25 to Form RL Structures in Plant Cells Is Necessary for Its Function in Movement, but Not for Its Suppression of RNA Silencing

**DOI:** 10.1371/journal.pone.0043242

**Published:** 2012-08-16

**Authors:** Fei Yan, Yuwen Lu, Lin Lin, Hongying Zheng, Jianping Chen

**Affiliations:** 1 State Key Laboratory Breeding Base for Zhejiang Sustainable Pest and Disease Control, Zhejiang Academy of Agricultural Sciences, Hangzhou, China; 2 Key Laboratory of Biotechnology in Plant Protection (Ministry of China), Zhejiang Academy of Agricultural Sciences, Hangzhou, China; 3 Institute of Virology and Biotechnology, Zhejiang Academy of Agricultural Sciences, Hangzhou, China; Virginia Tech, United States of America

## Abstract

The p25 triple gene block protein of *Potato virus X* (PVX) is multifunctional, participating in viral movement and acting as a suppressor of RNA silencing. The cell-to-cell movement of PVX is known to depend on the suppression function of p25. GFP-fused p25 accumulates in rod-like (RL) structures with intense fluorescence in cells. By monitoring the location of fluorescence at different times, we have now shown that the RL structure is composed of filaments. P25 mutants without the conditional ability to recover movement function could not form RL structures while the mutants that had the ability did form the structure, suggesting that the ability of p25 to form RL structures is necessary for its function in cell-to-cell movement, but not for its suppressor function. Moreover, chemical inhibition of microfilaments in cells destroyed the formation of the complete RL structure. Additionally, TGBp2 and TGBp3 were recruited into the RL structure, suggesting a relationship between the TGBps in virus movement.

## Introduction

The genome of *Potato virus X* (PVX, genus *Potexvirus*) has five open reading frames, three of which overlap and are termed the triple gene block (TGB). The three TGB proteins have molecular masses of 25, 12, and 8 kDa. TGBp1 (p25) is required for virus cell-to-cell movement. Studies using microinjection and biolistic bombardment have shown that p25 increases the size exclusion limit (SEL) of plasmodesmata (PD) and chaperones viral RNA and coat protein (CP) across PD [1], [2], [3], [4]. TGBp2 and TGBp3 also participate in, but are not sufficient for, viral movement [5], [6], [7], [8]. A recent article reviewed the movement strategies employed by TGB-encoding viruses and proposed models for viruses in the genera *Potexvirus*, *Hordeivirus*, and *Pomovirus* [9]. In these models, there are roles for the TGB proteins, coat protein and RNAs of the virus and also for the microfilaments, endoplasmic reticulum (ER), Golgi and PD of the host cell [9].

p25 is a multifunctional protein that also acts as a suppressor of RNA silencing [10] by affecting RDR6, a key component of the RNA silencing mechanism [11], [12], [13]. It has recently been shown that p25 interacts with Argonaute1 (Ago1), another central component of the mechanism, and mediates its degradation, probably indicating that p25 can suppress the plant RNA silencing mechanism by degrading Ago1 [14]. Analyses of virus mutants have shown that PVX movement is dependent on the suppression function of p25 but that suppression of silencing is not sufficient to allow virus movement between cells [15]. It is probable that other known properties of p25, such as its ATPase activity and its interaction with CP and cellular features, are also involved in virus movement.

In PVX-infected plant cells, GFP-fused p25 localizes to the nucleus, PD and cytoplasm where intensely fluorescent rod-like (RL) inclusions are seen [16]. When GFP-fused p25 is expressed alone under the control of the *Cauliflower mosaic virus* (CaMV) 35S promoter, the rod-like structures are still seen but p25 does not localize to PD, suggesting that other viral factors are needed for PD localization of p25 [16]. Research with the potexvirus *Bamboo mosaic virus* (BaMV) suggests that the RL structure is an active pool of TGBp1 but its role is unclear [3]. TGBp2 and TGBp3 are ER-associated proteins that co-localize [7], [8], [16]. When p25 and TGBp2 are co-expressed, ER-derived TGBp2 vesicles are seen along p25-labeled strands of cytoplasm [16]. When p25 and TGBp3 are co-expressed, the proteins seem to be closely associated [16].

We have investigated the formation of p25 RL structures and now report that the RL structure is composed of filaments. We have also provided evidence that the RL structure is necessary for the movement function of p25, but not for its ability to suppress RNA silencing. TGBp2 and TGBp3 were recruited into the RL structure, suggesting a joint involvement of the TGBps in virus movement.

## Results

### Formation of p25 Rod-like Structures in *Nicotiana benthamiana* Cells

**Figure 1:**
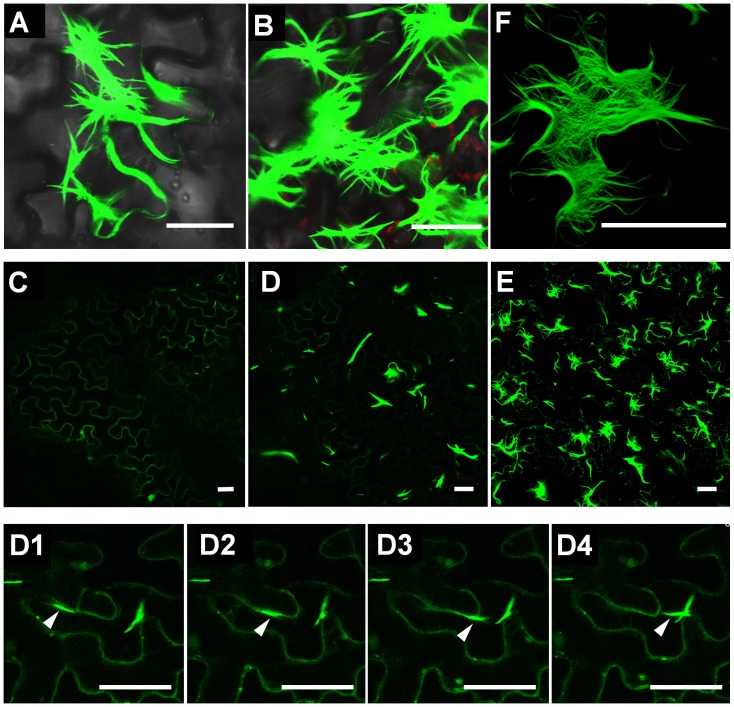
Rod-like structure formed by GFP-fused p25 (p25-GFP). A and B show the RL structure in *Nicotiana benthamiana* epidermal cells formed by p25-GFP expressed respectively from PVX-Δp25 and by agrobacterium infiltration. C, D and E show the location of p25-GFP expressed in cells at 1, 2 and 3 dpi, respectively. D1–D4 show a single moving fluorescent rod approaching a stationary one and merging with it at 2 dpi. F is a combined figure from a series of focal planes, showing that the RL structure is composed of thin filaments. Scale bar, 50 µm.

It has previously been reported [16] that expressed p25 fused with GFP at its N-terminus (GFP-p25) formed rod-like structures in both PVX-infected and uninfected cells. In our experiments, the structures also occurred when GFP fused with p25 at its C-terminus (p25-GFP) was expressed either alone under the control of CaMV 35S promoter or from the PVX-Δp25 vector with the duplicated coat protein subgenomic promoter (Fig. 1A and B). To study the process of formation of the structure, we expressed p25-GFP by agroinfiltration and detected the subcellular distribution of fluorescence 1, 2 and 3 days post infiltration (dpi) under the confocal microscope. At 1 dpi, p25-GFP fluorescence was distributed evenly at the cell periphery and there were also a few sporadic spots in the cytoplasm (Fig. 1C). At 2 dpi, the fluorescence on the cell periphery had diminished, while single fluorescent rods with intense fluorescence had started to appear (Fig. 1D). These rods moved within cells; a series of photos shows one fluorescent rod approaching and merging with a stationary one at 2 dpi (Fig. 1 D1–4). At 3 dpi, the fluorescence on the cell periphery was barely detected, while the RL structure was clearly formed (Fig. 1E). These observations suggest that the RL structure is progressively assembled from p25 protein.

### The Rod-like Structure is Composed of Filaments

The structure formed from GFP-fused p25 always showed intense fluorescence, and appeared to be composed of thick fluorescent rods when examined at a single focal plane under confocal microscopy. To examine the structure in detail, we decreased the strength of the exciting laser and monitored and recorded the fluorescence at a serial of focal planes and then combined them together. The combined figure showed that the structure was in fact composed of thin filaments (Fig. 1F) even though it looked like a thick rod when it was monitored at a single focal plane with a strong exciting laser. This is consistent with earlier reports from electron microscopy that the p25 formed “beaded sheets” [17].

### The Ability to Form the RL Structure is Necessary for p25 Movement but not for its Suppressor Function

**Figure 2:**
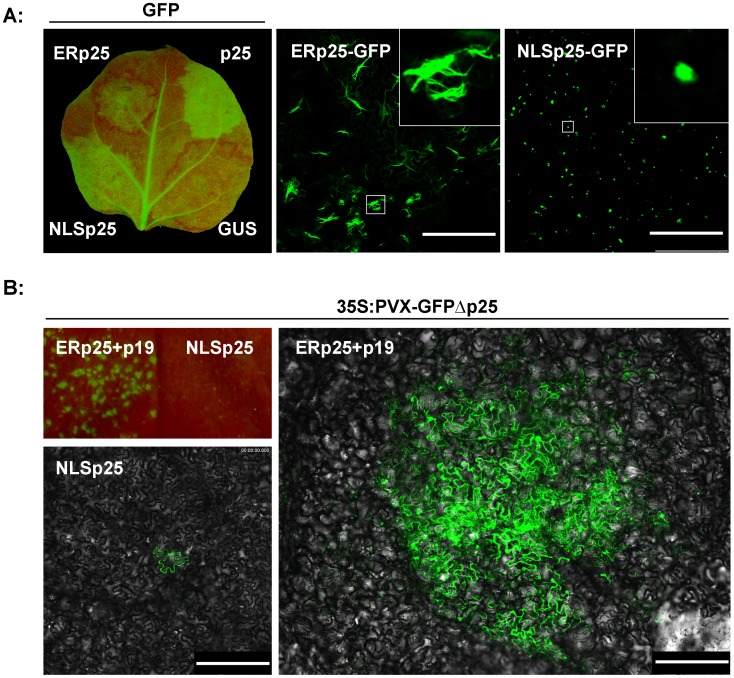
ERp25 loses its suppression function but can recover the cell-to-cell movement of PVX-GFPΔp25 when co-expressed with the heterologous silencing suppressor p19, while the opposite occurs with NLSp25. Panel A shows the suppression function analysis and localization of p25 fused to either the ER location signal or the NLS. NLSp25, but not ERp25, suppresses RNA silencing of GFP when co-expressed with GFP. When fused with GFP, ERp25 still formed an RL structure (although the structure is not entirely typical), but NLSp25 did not. White boxes show the locally enlarged regions. Panel B shows the ability of NLSp25 and ERp25 to recover cell-to-cell movement of the mutant virus PVX-GFPΔp25. Under long wave UV, fluorescent speckles are visible in the zones co-expressing ERp25 plus p19 and PVX-GFPΔp25 at 3 dpi, but not in those co-expressing NLSp25 and PVX-GFPΔp25 (top left panel). In the confocal micrographs, the fluorescence is limited to single epidermal cells when NLSp25 and PVX-GFPΔp25 are co-expressed (bottom left panel), but the fluorescence diffuses into the neighboring cells when ERp25 and PVX-GFPΔp25 are co-expressed together with p19 (right panel). Scale bar, 250 µm.

To determine whether the RL structure is associated with the movement or suppression functions of p25, we attempted to interfere with the formation of the RL structure by fusing an ER or NLS location signal at its amino terminal. NLSp25 expressed by agrobacterium remained a silencing suppressor but did not form an RL structure (Fig. 2A). In contrast, ERp25 could still form an RL structure (although its structure was not perfect) but its suppression of RNA silencing was greatly diminished (Fig. 2A, Fig. S1). Furthermore, ERp25, but not NLSp25, recovered the cell-to-cell movement of PVX-GFPΔp25 with the heterologous silencing suppressor p19 (Fig. 2B; the average diameter of thirty infection loci was about 700 µm). These results imply that the ability of p25 to form the RL structure is necessary for its cell-to-cell movement function, but not for its ability to act as a suppressor.

**Figure 3:**
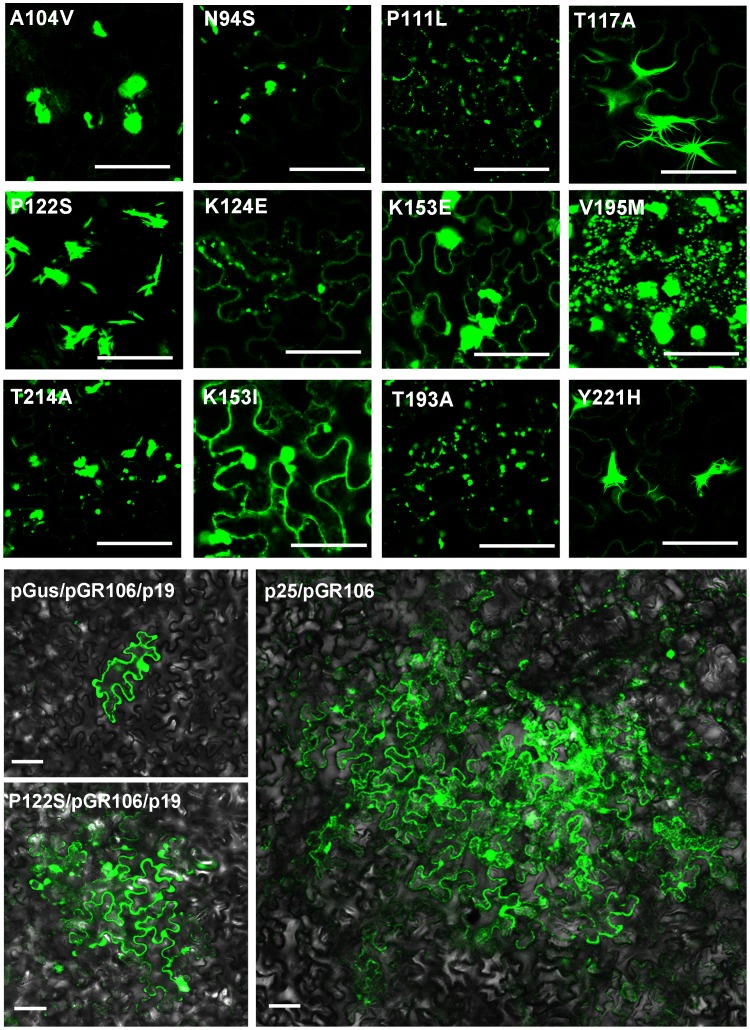
Investigating the RL structure of the reported p25 mutants. Upper panels: twelve single amino acid mutants of p25 were fused with GFP and expressed in epidermal cells. An RL structure is visible in cells expressing T117A, P122S or Y221H fused with GFP, but not in cells expressing the other GFP-fused mutants. Lower panels: Recovery analysis of viral movement with mutant P122S. Fluorescence diffuses into the neighboring cells in nearly all the loci when P122S and PVX-GFPΔp25 (pGR106) are co-expressed together with p19 (bottom left), suggesting that P122S can recover the movement of PVX-GFPΔp25 when co-expressed with the suppressor p19. The recovery is weak compared to the wild-type control (p25/pGR106, bottom right) but nevertheless helps virus move into limited layers of neighboring cells (average about 200 µm) compared to the negative control (pGus/pGR106/p19). Scale bar, 50 µm.

To confirm these findings, twelve single amino acid mutants of p25 reported previously were used for analysis [15]. Eleven of the mutants, N94S, P111L, T117A, P122S, K124E, K153E, K153I, T193A, V195M, T214A and Y221H, are deficient in both virus movement and silencing suppression (double functional deficient or DFD mutants), and of these T117A and Y221H are reported to recover the cell-to-cell movement of PVX-GFPΔp25 when co-expressed with another suppressor. The twelfth mutant, A104V, is deficient in movement but not in suppression. We examined the RL structure of each mutant fused with GFP in agrobacterium-infiltrated cells. The RL structure was not formed in cells expressing GFP-fused A104V, which supported the view that formation of the structure was not necessary for p25 to act as a suppressor (Fig. 3). Among the DFD mutants, typical RL structures were seen where GFP was fused to T117A or Y221H, some slender RL structures were seen with P122S, but there were no structures with the other mutants (Fig. 3). Thus, with the exception of P122S, only those DFD mutants with the conditional ability to recover movement function formed the RL structures. This gives further support to the conclusion that the ability of p25 to form the RL structure is necessary for its function in cell-to-cell movement but not for its ability to act as a suppressor (Fig. 3). We then re-investigated the reported inability of mutant P122S to recover the movement of PVX-GFPΔp25 when co-expressed with another suppressor. Results from more than three repeats showed that when P122S was co-expressed with p19, fluorescence was visible in 6–10 cells at almost all loci whereas in the negative control it was limited to a single cell. This indicated that P122S could indeed recover the movement of PVX-GFPΔp25 when co-expressed with the suppressor p19, although the recovery was weak and the helper virus moved only into limited layers of neighboring cells (Fig. 3; the average diameter of thirty infection loci was about 200 µm, compared to the positive control of about 700 µm). Hence, the result from P122S also supported the conclusion above.

### Formation of RL Structures is Inhibited by LatB

**Figure 4:**
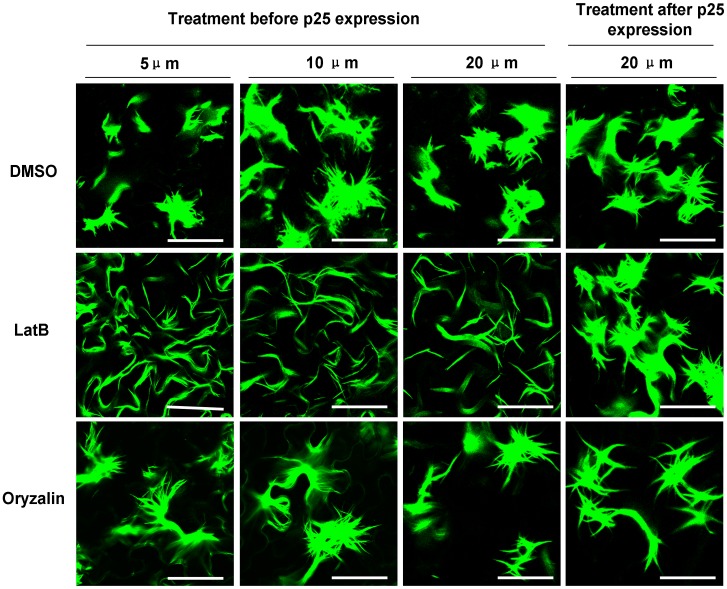
Effect of chemical inhibition of microfilaments or microtubules on the formation of the RL structure. At 5–20 µM, LatB, an inhibitor of microfilaments, inhibits the formation of the complete RL structure, but not the formation of primary RL filaments. After formation of the RL structure, LatB treatment has no effect. Treatment with Oryzalin, an inhibitor of microtubules, has no effect on the formation of the structure either before or after the structure is formed. Scale bar, 50 µm.

The microfilaments and microtubules that form the host cytoskeleton are implicated in the movement of both plant and animal viruses. It has been reported that treatment with the microfilament inhibitor latrunculin B (LatB) severely limited the spread of PVX in plants [18]. Since the RL structure is necessary for PVX movement, we next examined the relationship between microfilaments and RL structures. Tobacco epidermal cells were treated with different concentrations of LatB (5 µM, 10 µM and 20 µM) for 3 h before infiltration with the agrobacterium containing the vector expressing GFP-fused p25. At 3 dpi, the treated cells were examined under the confocal microscope. No complete RL structures, but only single fluorescent filaments, were seen in cells treated with LatB at any concentration, while the entire normal-looking RL structure was seen in the control cells treated with DMSO (Fig. 4). The RL structure was not affected by treatment with LatB 3 days after p25 had been expressed, showing that microfilaments were necessary during the formation of the RL structures, but not after they were formed. The microtubule inhibitor oryzalin did not affect the formation of the structure, whether treated before or after p25 expression (Fig. 4) suggesting that microtubules are unnecessary for RL structure formation.

### TGBp2 and TGBp3 are Recruited to the RL Structure

**Figure 5:**
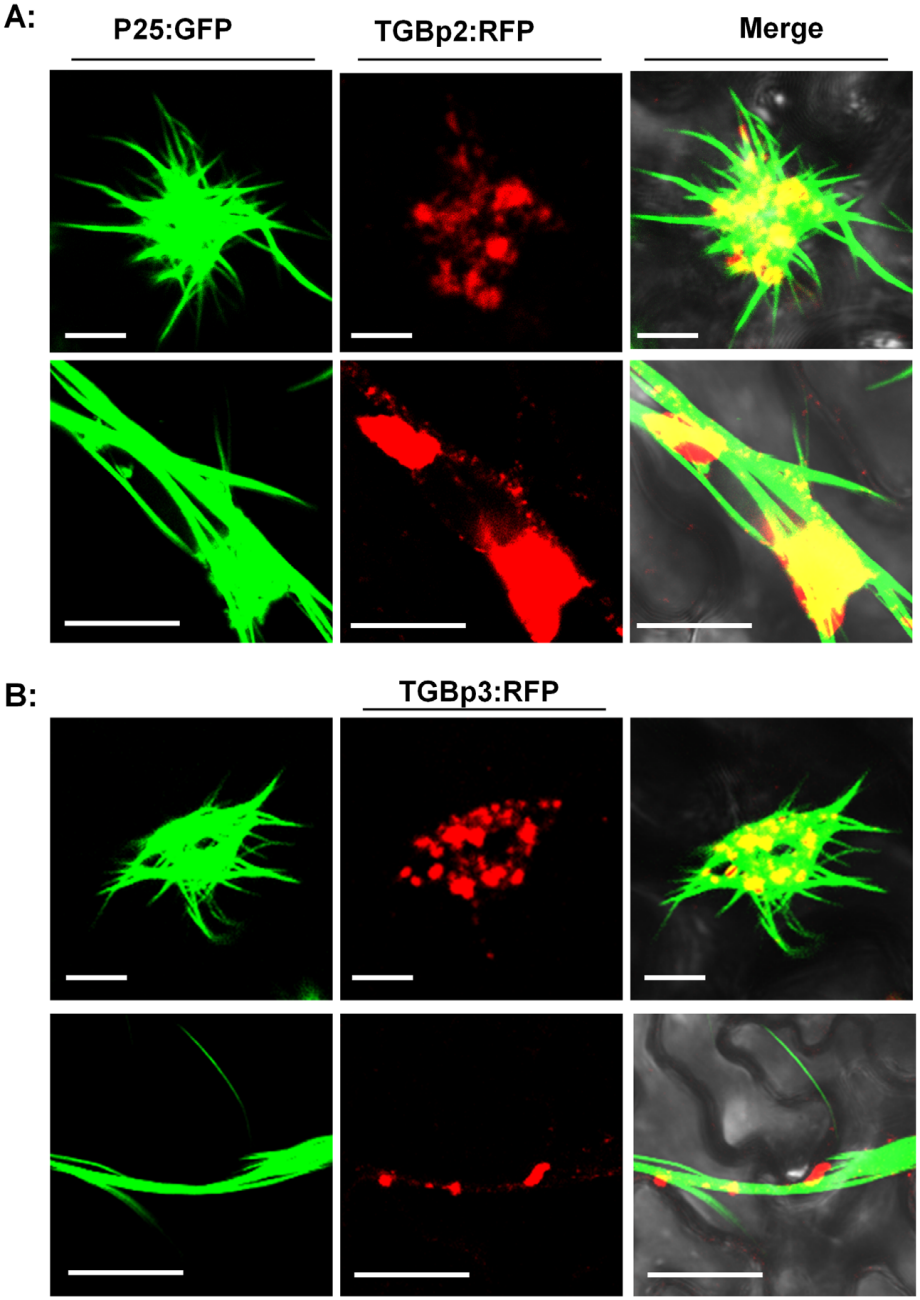
TGBp2 and TGBp3 are aligned on the RL structure. A shows the co-location of RFP fused-TGBp2 and GFP-fused p25 at different scales. B shows the co-location of RFP fused-TGBp3 and GFP-fused p25 at different scales. Scale bar, 20 µm.

**Figure 6:**
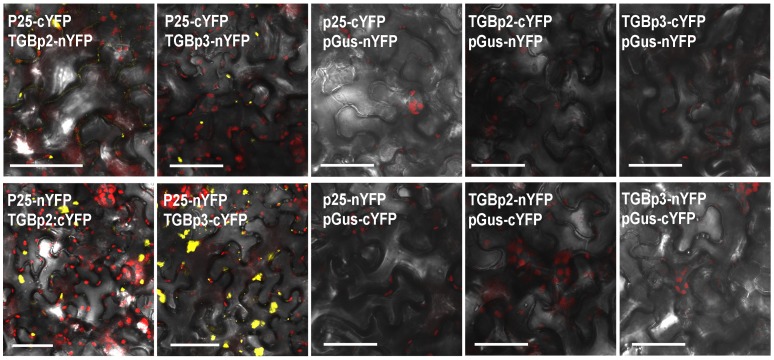
Interaction of TGBp2 and TGBp3 with p25 in BiFC assay. Scale bar, 20 µm.

Since both TGBp2 and TGBp3 are known to be necessary for PVX movement, we fused them individually with RFP and co-expressed them with GFP-fused p25 in tobacco epidermal cells by agroinfiltration. Examination of the fluorescence 3 dpi showed that both RFP-fused TGBp2 and TGBp3 formed granules as described in previous reports and that almost all these granules were recruited into the RL structure and not merely attached to the outside (Fig. 5A, B). At high resolution, it could be seen that red granules aligned on the filaments of the RL structure, hinting that p25 was associated with both TGBp2 and TGBp3 (Fig. 5 A, B). Because no detectable interactions have been reported between these proteins in previously reported yeast two-hybrid (YTH) experiments [16], we then used bimolecular fluorescence complementation analysis (BiFC) to analyze the interactions between p25 and TGBp2 or TGBp3. BiFC is a powerful tool for studying protein-protein interactions in living cells, and its results are better than YTH in reflecting natural interactions. Interactions between TGBp1 and TGBp2 of the potexvirus BaMV have previously been reported using this technique [19]. Here, yellow fluorescence (recorded as green during imaging) was seen at 3 dpi in the cells co-expressing pCV-n(c)YFP-p25 and either pCV-c(n)YFP-TGBp2 or pCV-c(n)YFP-TGBp3 (Fig. 6), but not in the control cells co-expressing pCV-n(c)YFP-p25 and pCV-c(n)YFP-pΔGUS, or pCV-c(n)YFP-TGBp2 and pCV-n(c)YFP-pΔGUS, or pCV-c(n)YFP-TGBp3 and pCV-n(c)YFP-pΔGUS (Fig. 6). This suggests that there are indeed interactions between p25 and both TGBp2 and TGBp3 in plant cells.

## Discussion

Rod-like inclusions were first found associated with p25 by electron microscopy of PVX-infected tissues [3], [17], [20]. Subsequently, Samuels et al. used confocal microscopy to observe the typical rod-like structures in both PVX-infected and p25-expressing epidermal cells [16]. We have now shown that the rod-like structure is actually composed of interweaved filaments. This may have been overlooked because GFP-fused p25 is usually expressed at high levels because of its suppression function, and so appeared rod-like.

In our experiments, p25 was first expressed generally in cells (the first day) and by the second day had assembled into the primary RL filaments. These were then recruited into the complete structure by the third day. This process must require self-interaction of p25, which has been experimentally demonstrated in several experiments, satisfying the requirement of the model [16], [21]. Previous studies with virus mutants have shown that the movement and suppression functions of p25 are separate properties, and that suppression is a precondition, but is not sufficient for, movement [15]. In our studies, only those mutants that could form the RL structure were able to recover the movement of p25-deficient PVX, suggesting that the ability to form the RL structure may also be a precondition for the movement function of p25.

Microfilaments are known to be essential for PVX cell-to-cell movement. Vesicles containing GFP-TGBp2 protein have been seen adjacent to microfilaments in plant cells, and treatment with LatB caused dispersal of these vesicles [18]. Our studies show that microfilaments are also necessary for the formation of the RL structures particularly at the stage where the filaments are recruited into the complete structure (Fig. 3). A recent paper reported that PVX p25 reorganized the actin cytoskeleton [22]. Both results therefore show a relationship between p25 and microfilaments during PVX movement.

Both TGBp2 and TGBp3 are known to be necessary and to function together with p25 for cell-to-cell movement of PVX. GFP-fused TGBp2 forms ER-derived granular vesicles that are necessary for virus movement and which can be seen alongside the p25-formed strands in the cytoplasm [7], [8], [16]. GFP-fused TGBp3 is mainly located in the ER network when expressed alone [5], [7], but has a similar location to TGBp2 and co-localizes with p25 in the nucleus when expressed with PVX [16], [23]. It is thought that TGBp2 may direct TGBp3 into the same ER-derived vesicles during virus infection [4]. Consistent with a recent paper that reports the recruitment of TGBp2 and TGBp3 to the TGBp1 aggregates [22], we here present evidence that PVX TGBp2 and TGBp3 granules align on the filaments of the p25 RL structure (Fig. 5). Our BiFC results also demonstrate an *in vivo* interaction between p25 and both TGBp2 and TGBp3 (Fig. 6). The results therefore suggest the possibility that TGBp2 and TGBp3 of PVX are recruited into the RL structure by interaction with p25 to enable viral movement.

In PVX-infected plant cells, the perinuclear whorl-like X-body is another inclusion structure. Tilsner *et al* (2012) recently reported that PVX TGBp1 organized the formation of the X-body by re-modeling host actin and endomembranes, and that TGBp2/3 were recruited to the structure. It was speculated that the X-body plays a role as a viral replication factory, although virus replication and assembly can proceed without it albeit with reduced efficiency [22]. Here, we show that the RL structure formed by p25 may participate in the cell-to-cell movement function of p25, but not in its suppressor function. Meanwhile, further research is needed to investigate the relationship between the p25-formed RL structure and the X-body.

## Materials and Methods

### Bacterial Strains and Plasmids

**Figure 7:**
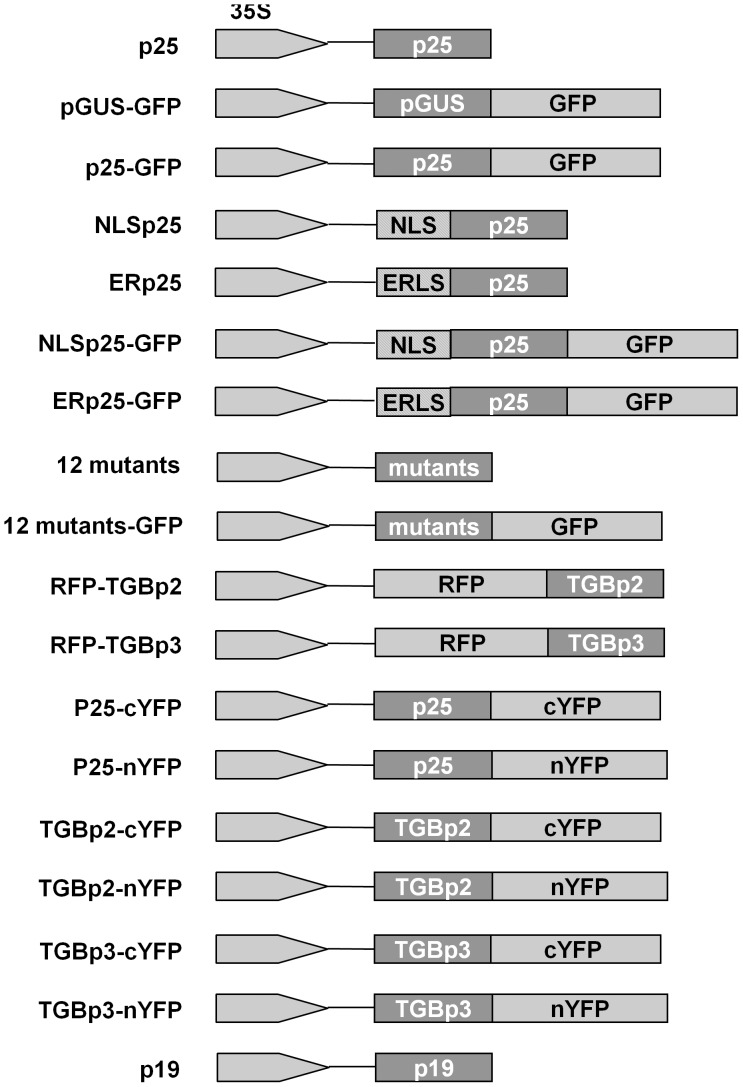
Diagram showing the plasmids used in this work. Plamsids were constructed into the pCV1300 binary vector that was developed from pCAMBIA1300 with the CaMV 35S promoter.

Construction of all plasmids followed standard cloning techniques. *Escherichia coli* strain TG1 was used for transformation [24]. All constructed plasmids are shown in Fig. 7. pCV:GFP, pCV:RFP were as described previously [25]. p25 which was introduced into pGEM-T (Promega, Madison, WI) with forward primer (5′-TCTAGAATGGATATTCTCATCAGTAGTT -3′, *Xba* I site underlined) and reverse primer (5′-GGATCCCTATGGCCCTGCGCGGA -3′, *Bam*H I site underlined), was digested with Xba I and *Bam*H I, and ligated into the pCV:GFP and digested with the same enzymes to generate pCV:p25-GFP. pCV: RFP-TGBp2 and pCV: RFP-TGBp3 were produced with primer pairs (5′-GGATCCATGTCCGCGCAGGGCCATA-3′, *Bam*H I site underlined, 5′-GAGCTCCTAATGACTGCTATGATTGTC-3′, *Sac* I site underlined) and (5′-GGATCCATGGAAGTAAATACATATCTCA-3′, *Bam*H I site underlined 5′-GAGCTCTCAATGGAAACTTAACCGTTC-3′ *Sac* I site underlined), respectively. To express p25-GFP in a virus vector, the pGR106-Δp25 vector was used. p25-GFP was amplified from pCV:p25-GFP with primer pair 5′-ATCGATATGGATATTCTCATCAGTAGTT-3′ (*Cla* I site underlined) and 5′-GTCGACTCACTTGTACAGCTCGTCC-3′ (*Sal* I site underlined), then ligated into the pGR106-Δp25 digested with the same enzymes to generate pGR106-Δp25- p25-GFP.

The BiFC assay used pCV:cYFP and pCV:nYFP [25]. The same primers and restriction sites listed above were used in constructing the vectors of p25, TGBp2 and TGBp3. The p25 mutants were obtained by PCR according to the mutant sequences reported [15]. The ER location site (ATGAAGACTAATCTTTTTCTCTTTCTCATCTTTTCACTTCTCCTATCATTATCCTCGGCCGAA) used in previous reports [26] and the widely-used NLS of *Simian virus* 40 large T antigen (ATGCCTCCAAAAAAGAAGAGAAAGGTC) were used for fusion PCR to produce vectors expressing ERp25 and NLSp25.

### Plant Material and Agrobacterium Infiltration

Subcellular targeting of proteins by fluorescence was explored in *Nicotiana benthamiana*. *N. benthamiana* line 16 c was used to analyse gene silencing suppression as previously reported [10]. Briefly, agrobacterium mixtures carrying 35S-green fluorescent protein (35S-GFP) and the individual constructs were infiltrated into leaves of 16c plants. GFP fluorescence was viewed under long-wavelength UV light 5 dpi. Agrobacterium strains C58C1 and GV3101 were used for agrobacterium infiltration at OD_600_ = 1.0 except where stated. Equal volumes of individual agrobacterium cultures (OD_600_ = 1.0) were mixed before co-infiltration. The analysis using chemical inhibition of the cytoskeleton was carried out as reported [18].

### Microscopy

The Leica TCS SP5 (Leica Microsystems, Bannockburn, IL) confocal laser scanning microscope system was used to examine the fluorescence of GFP, RFP and YFP. Unless otherwise stated, fluorescence was monitored at 5 dpi. GFP was excited at 488 nm and the emitted light captured at 505–525 nm; RFP was excited using 543 nm and captured at 590–630 nm; YFP was excited at 514 nm and captured at 555–575 nm. All images were processed using Adobe Photoshop version 7.0 software (Adobe Systems Inc., San Jose, CA).

### Recovery Experiments Using p25 Mutants

PVX movement recovery experiments were carried out according to previous reports [15]. Briefly, an agrobacterium culture GV3101 with the 35S:PVX-GFPΔp25 (pGR106) was diluted 10,000-fold, then mixed 1∶1∶1 with agrobacterium culture C58C1 carrying 35S:p19 and 35S:p25 mutants.

## Supporting Information

Figure S1 **Northern blot showing that NLSp25, but not ERp25, retains the wild-type ability of PVX p25 to suppress RNA silencing.** *gfp* mRNAs in infiltrated zones (shown in Fig. 2A) were hybridized with a GFP DNA probe.
(TIF)
