# Supplementary figures and images for: The Ability of PVX p25 to Form RL Structures in Plant Cells Is Necessary for Its Function in Movement, but Not for Its Suppression of RNA Silencing
Source: PLoS One. 2012 Aug 16;7(8):e43242. doi: 10.1371/journal.pone.0043242 (PMC3420909; doi:10.1371/journal.pone.0043242)

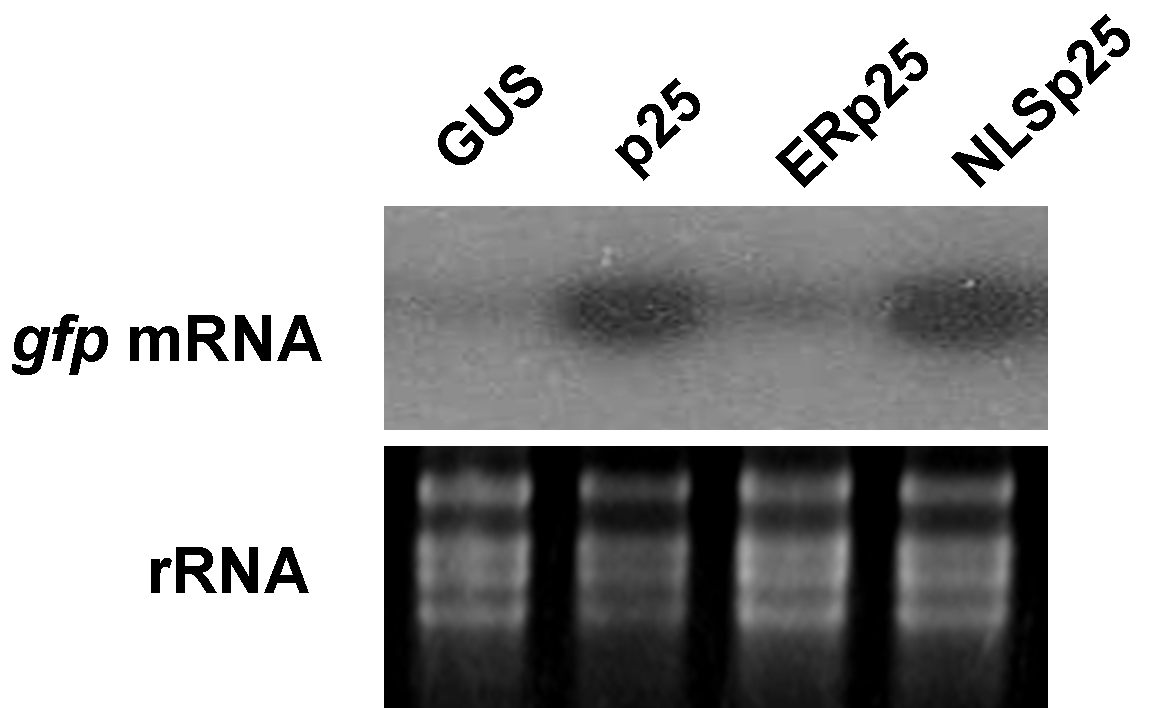

Supplement: Figure S1 — Northern blot showing that NLSp25, but not ERp25, retains the wild-type ability of PVX p25 to suppress RNA silencing. gfp mRNAs in infiltrated zones (shown in Fig. 2A) were hybridized with a GFP DNA probe. (TIF) [file pone.0043242.s001.tif]
